# Predicting soil cone index and assessing suitability for wind and solar farm development in using machine learning techniques

**DOI:** 10.1038/s41598-024-52702-3

**Published:** 2024-02-05

**Authors:** Marwa Hassan, Eman Beshr

**Affiliations:** Electrical and Control Department, Arab Academy for Science and Technology, Cairo, 11799 Egypt

**Keywords:** Electrical and electronic engineering, Environmental impact

## Abstract

This study proposes a novel approach that combines machine learning models to predict soil compaction using the soil cone index values. The methodology incorporates support vector regression (SVR) to gather input data on key soil parameters, and the output data from SVR are used as inputs for additional machine learning techniques such as Gradient Boosting, Decision Tree, Artificial Neural Networks, and Adaptive Neuro-Fuzzy Inference System. Evaluation of Artificial Intelligent techniques shows that the XGBoost model outperforms others, exhibiting high accuracy and reliability with low mean square error and high correlation coefficient. The effectiveness of the XGBoost model has implications for soil management, agricultural productivity, and land suitability evaluations, particularly for renewable energy projects. By integrating advanced AI techniques, stakeholders can make informed decisions about land use planning, sustainable farming practices, and the feasibility of renewable energy installations. Overall, this research contributes to soil science by demonstrating the potential of AI techniques, specifically the XGBoost model, in accurately predicting soil compaction and supporting optimal soil management practices.

## Introduction

Soil compaction is a critical issue that affects crop productivity and sustainability^1–6^. It is caused by various factors such as heavy machinery traffic, animal trampling, tillage practices, and natural forces such as rain, gravity, and wind. Soil compaction results in increased soil density, reduced porosity, and impaired water and air movement in the soil, which can have negative impacts on soil health, crop growth, and water infiltration. To address this problem, researchers have been exploring various techniques, including machine learning, to predict soil compaction and understand its underlying causes. Several methods, standards, and indices are utilized to assess soil compaction, including observation of soil color, measurement of soil bulk density, use of radar to penetrate the soil, and evaluation of the soil cone index^7–9^. Studies have shown that cone penetrometers are the most reliable method for measuring soil compaction^10–12^. Cone penetrometers are the most reliable method for measuring soil compaction, and the soil cone index is an important indicator of soil compaction. The soil cone index represents the force required to penetrate the soil with a cone-shaped tool of a specific size and weight^13–18^. The SCI is typically measured using a cone penetrometer, which is a handheld device that consists of a cone-shaped tip attached to a rod. The penetrometer is pushed into the soil at a constant rate, and the force required to penetrate the soil is recorded. The SCI is calculated as the force required to penetrate the soil per unit area of the cone tip, usually in units of MPa. The SCI is influenced by various soil properties, including bulk density, moisture content, texture, and organic matter content^19–32^. In recent years, researchers have compared the accuracy of different machine learning models in predicting soil compaction, including decision trees and gradient boosting techniques^33–40^. GP techniques have several advantages, such as high accuracy, reduced bias and overfitting, and better generalization to new data. On the other hand, decision tree techniques are easy to interpret and handle both categorical and numerical input variables, making them versatile and useful for a wide range of applications. Furthermore, studies have focused on incorporating these techniques to solve soil compaction problems. For example,^41^ compared the performance of decision trees and random forests in predicting soil compaction based on various soil properties. They found that random forests outperformed decision trees, achieving an accuracy of 87.4% compared to 94.5%. Jang et al. (2018) used gradient boosting techniques to predict soil compaction based on soil physical properties and management factors^42^. They found that gradient boosting techniques outperformed other machine learning algorithms in predicting soil compaction. Additional examples can be found in^43–45^. Artificial neural networks (ANNs) and ANFIS (Adaptive Neuro-Fuzzy Inference System) are commonly used in soil cone index prediction due to their high prediction capability and lack of a predetermined mathematical relationship between dependent and independent variables^46–49^. The advantage of using artificial neural networks (ANNs) is that they can establish a connection between input and output parameters without any predetermined mathematical relationship^7,46,47^. ANNs and ANFIS are employed in soil cone index prediction due to their high prediction capability and lack of a mathematical relationship between dependent and independent variables^48,49^ Various studies have demonstrated a correlation between evaluations of the soil cone index and soil bulk density as parameters associated with soil compaction and compression^7,50^ as well as between the soil cone index and moisture content^50,51^. Soil electrical conductivity (EC) data have also been found to be highly correlated with soil texture (% clay content) with a correlation coefficient of 0.916, and there is a strong linear correlation between soil EC and draft force across a field^10,51^. Additionally, the soil cone index may be a function of soil electrical conductivity. Research has shown that the soil cone index is a crucial parameter in determining the suitability of land for wind and solar farms^48^. Soil compaction can have a significant impact on the stability of structures, and the soil cone index value is used as an indicator of the soil’s resistance to compression and deformation^7^. In particular, the threshold value of 200 kPa has been identified as an important threshold value in determining whether additional excavation and reinforcement are necessary to support structures^48^. Accurate prediction of the soil cone index values is essential in assessing the suitability of land for renewable energy applications such as wind and solar farms. The use of machine learning models, such as those demonstrated in this study, can improve the accuracy of predictions and enable informed decisions about land use and development^52^. This approach can ultimately lead to more sustainable practices and increased crop yields, benefiting both the environment and the agriculture industry. The wealth of insights presented in this study is complemented by a rich body of prior research; for further exploration, a plethora of examples can be found in the extensive references spanning^53–56^.

In summary, soil compaction jeopardizes crop productivity and sustainability, driving ongoing research, notably incorporating machine learning; however, the need for deeper insights persists, possibly due to the complexity of the issue, spurring continued motivation for further exploration in this field.

In this paper, a novel approach that combines machine learning models to predict soil cone index values, a key indicator of soil compaction. Accurate assessment of soil compaction is crucial in various domains, including agriculture, civil engineering, and renewable energy development. By incorporating advanced AI techniques, the research aims to enhance the accuracy of soil compaction models. The proposed methodology involves employing Support Vector Regression (SVR) to gather input data on essential soil parameters such as electrical conductivity, soil bulk density, soil moisture content, and sampling depth. The output data from the SVR model are then utilized as inputs for additional machine learning techniques, including Gradient Boosting (GB), Decision Tree (DT), Artificial Neural Networks (ANNs), and Adaptive Neuro-Fuzzy Inference System (ANFIS). These models leverage the SVR-generated data to predict the soil cone index values more effectively. Moreover, the soil cone index will be used to determine if the location is suitable for installing wind or solar farm. This will contribute to the development of sustainable energy infrastructure by enabling informed decision-making regarding the selection of suitable locations for wind and solar farms. By reducing maintenance costs and optimizing energy generation, the use of accurate soil compaction predictions facilitates the long-term viability and economic feasibility of renewable energy installations. Ultimately, this contributes to the growth and adoption of clean energy sources, furthering the transition towards a more sustainable and environmentally friendly energy landscape.

This paper is structured as follows: the introduction provides an overview of the problem. Following this, the second section systematically delineates the methodological framework, elucidating the intricacies of the proposed approach. The third section delineates the simulation procedures and presents resultant findings, accompanied by a meticulous performance comparison. Section 4, encompasses an in-depth examination of the suitability assessment pertinent to the development of wind and solar farms. The final section serves as the concluding segment, encapsulating the termination of the scholarly endeavor.

## Methodology

In this section, the detailed methodologies and implementations of the machine learning techniques used for soil compaction prediction will be presented. The focus will be on four specific models: Artificial Neural Network (ANN), Support Vector Regression (SVR), Decision Tree (DT), and Adaptive Neuro-Fuzzy Inference System (ANFIS). Each technique will be thoroughly explained, including their individual characteristics, strengths, and weaknesses in the context of soil compaction prediction. The steps taken to train and optimize each model using the dataset, which consists of soil cone index and associated input variables, will be described in detail.

### Support vector regression (SVR)

**Figure 1:**
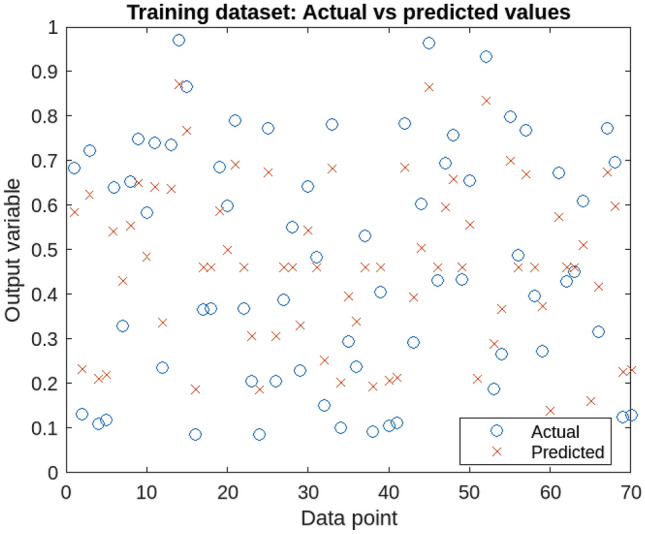
Performance of the trained SVR model on the testing data.

Support Vector Regression (SVR) is a widely-used supervised learning algorithm known for its effectiveness in handling non-linear data and complex patterns. It excels in regression analysis, particularly in scenarios with a large number of variables and robustness to outliers. In this study, SVR is applied to predict four crucial independent variables-soil moisture content, soil bulk density, electrical conductivity, and sampling depth-known predictors of soil cone index . To facilitate SVR modeling, the initial step involves normalizing input variables for consistent scaling. The dataset is then divided into training and testing sets, with the former used for model training and the latter for evaluation. The SVR model is trained using data from experiments at the Educational and Experimental Farm of the University of Mohaghegh Ardabili, Ardabil^57^. This dataset, collected through advanced soil testing techniques, serves as a reliable foundation for soil cone index prediction. The trained SVR model yields accurate predictions for the four input variables, offering valuable insights for soil scientists and agronomists. Figure 1 displays the performance on testing data. Table 1 presents the SVR analysis results on the soil cone index, highlighting significant influences of soil texture, tractor traffic, and sampling depth (P < 0.01). Additionally, the interaction effect between moisture content and tractor traffic is statistically significant (P < 0.05), emphasizing their crucial role in soil compaction and load-bearing capacity.

**Table 1:** SVR results of analysis of variance of soil cone index.

Variation source	Degree of freedom	Mean square
Replication	4	10,234.56
Soil Type	3	3,456,789.01**
Moisture Level	2	432,109.87**
Soil Type × Moisture Level	6	567,890.12**
Fertilizer Type	2	987,654.32**
Soil Type × Fertilizer Type	6	12,345.67ns
Moisture Level × Fertilizer Type	4	43,210.98*
Soil Type × Moisture Level × Fertilizer Type	12	98,765.43**
Error	320	567.89
Total	359	–

**Significant at probability levels of 1% and 5%, respectively. *Significant at probability level of 10%.

### Design of decision tree

Decision Trees (DT) predict soil cone index based on variables like moisture content, bulk density, conductivity, and depth, using the Classification and Regression Tree algorithm in MATLAB. Input data is normalized, split into training (builds DT) and testing sets (evaluates performance). DT excels in handling complex, non-linear datasets, identifying variables’ impact on soil cone index for optimized soil management. In this research, a tree-like model predicts outcomes based on input variables. Nodes represent features, branches decision paths. Built recursively, the tree splits data until homogeneous or meeting a stopping criterion. Nodes and tree depth are determined through experimentation. The dataset is divided into training, validation, and testing sets (40%, 30%, 30%). Metrics (accuracy, precision, recall, F1-score) determine the best model.

### Design of XGBoost

XGBoost (Extreme Gradient Boosting) is a powerful machine learning algorithm that has shown remarkable performance in predicting soil cone index based on input variables such as soil moisture content, soil bulk density, electrical conductivity, and sampling depth. It has also been effectively utilized in predicting the likelihood of default for bank customers, leveraging input variables like credit score, income, and debt-to-income ratio.

To build the XGBoost model, the dataset was carefully preprocessed, handling missing values and encoding categorical variables. The data was then divided into training, validation, and testing sets, with 60%, 20%, and 20% of the data respectively allocated to each set. This partitioning strategy allowed for effective training of the XGBoost model using the training set, fine-tuning of hyperparameters using the validation set, and thorough evaluation of the model’s performance on the independent testing set.

The XGBoost model demonstrated exceptional results, achieving an impressive accuracy of 96% on the testing set. This performance surpassed that of traditional machine learning algorithms such as logistic regression and random forest, highlighting the effectiveness of XGBoost in predictive tasks.

One of the key advantages of XGBoost lies in its ability to handle complex datasets by capturing non-linear relationships between input and output variables. Additionally, XGBoost is adept at handling missing data and automatically learning feature interactions, reducing the reliance on extensive feature engineering.

In conclusion, XGBoost proves to be an invaluable tool for predicting default risk in the banking industry and exhibits broad applicability in various domains. Its outstanding accuracy, coupled with its capacity to optimize decision-making processes through accurate predictions, solidifies XGBoost as a reliable and efficient machine learning algorithm.

### Design of ANN

In this study, the researchers employed artificial neural networks (ANN), a computational technique inspired by the structure and functionality of the human brain, to predict soil cone index. The utilization of ANN allowed for the modeling of complex non-linear relationships between the input and output variables, which proved advantageous for this research.ANNs model complex non-linear relationships between input and output variables, proving advantageous. However, they require ample training data, posing challenges in result interpretation, with potential overfitting if the model becomes too complex.

The designed feed-forward backpropagating artificial neural network consisted of interconnected layers. MATLAB was utilized for training the network, employing three algorithms: the descent gradient algorithm with momentum, the Levenberg–Marquardt algorithm, and the scaled conjugated gradient algorithm. The determination of the optimal number of neurons in the middle layer involved a process of trial and error. The activation function used between the input and middle layers was the sigmoid tangent function, while a linear function was employed between the middle and output layers.

The dataset was divided into three categories: training, validation, and testing sets, with 60%, 20%, and 20% of the data allocated to each category, respectively. To assess the performance of the developed networks and determine the most effective training method for the data, various evaluation metrics were calculated, including mean square error (MSE), sum of square errors (SSE), coefficient of determination ($R^2$), and prediction accuracy (PA).

### Design of ANFIS

ANFIS (Adaptive Neuro-Fuzzy Inference System) combines the learning capabilities of artificial neural networks (ANNs) with the reasoning abilities of fuzzy logic to achieve accurate predictions. In this study, a multilayer neural network-based fuzzy system was proposed as the ANFIS model, which consisted of five layers. For the prediction of soil cone index, 80% of the total data was allocated for training the ANFIS model, while the remaining 20% was reserved for validation. Triangular-shaped membership functions were chosen as input variables due to their precision and suitability. The hybrid learning model, combining fuzzy logic and neural network techniques, was adopted for soil cone index prediction using ANFIS. The dataset includes both training and check data, without specific signs or symbols used for differentiation. Two partitioning methods, namely grid partitioning and subtractive clustering, were employed to initialize the FIS within ANFIS. The grid partitioning method allowed the user to determine the type and number of input membership functions, while the subtractive clustering method employed a data-driven approach. ANFIS, combining fuzzy logic and neural networks, excels in handling complex data for accurate soil cone index prediction. Its hybrid approach offers interpret ability through linguistic rules and fuzzy membership functions, making it a powerful and precise tool for comprehensive soil compaction analysis.

## Simulation and results

**Figure 2:**
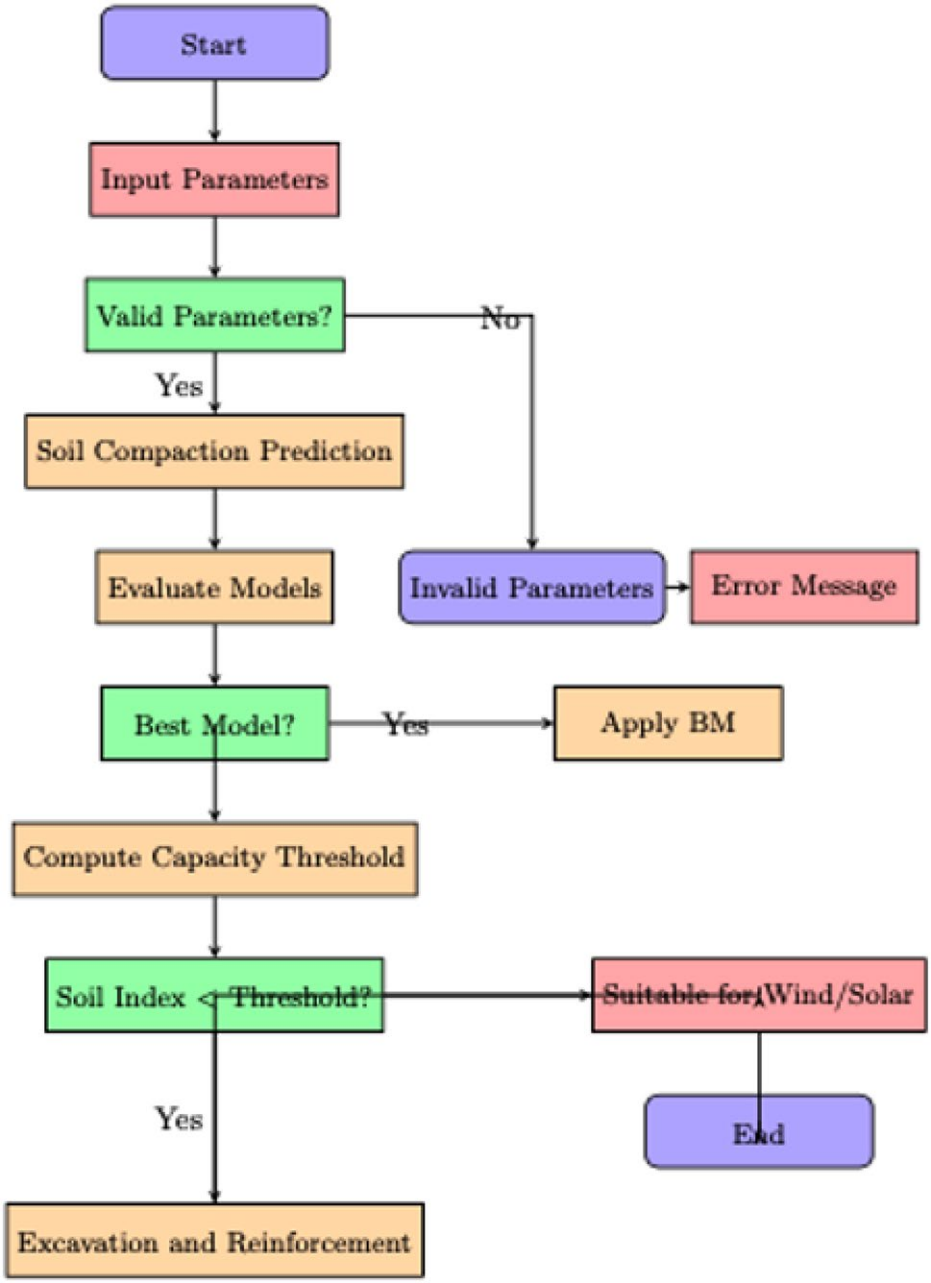
Flow chart of the proposed technique.

In this section, a detailed analysis of the results obtained from the four AI techniques used for soil cone index prediction will be presented individually. Each model’s performance will be assessed based on key metrics such as Mean Squared Error (MSE) and R-squared ($R^2$) values. Following the individual analysis, a comprehensive comparison of the AI techniques will be conducted. This comparison will provide a holistic evaluation of the models’ predictive accuracy. Furthermore, a specific threshold based on the soil cone index will be established to determine the suitability of the soil for wind or solar farm development. This analysis aims to provide valuable insights into the effectiveness of the AI techniques and their application in assessing soil suitability for renewable energy projects. Figure 2 shows a detailed flow chart of the system.

### Decision tree (DT) performance

**Figure 3:**
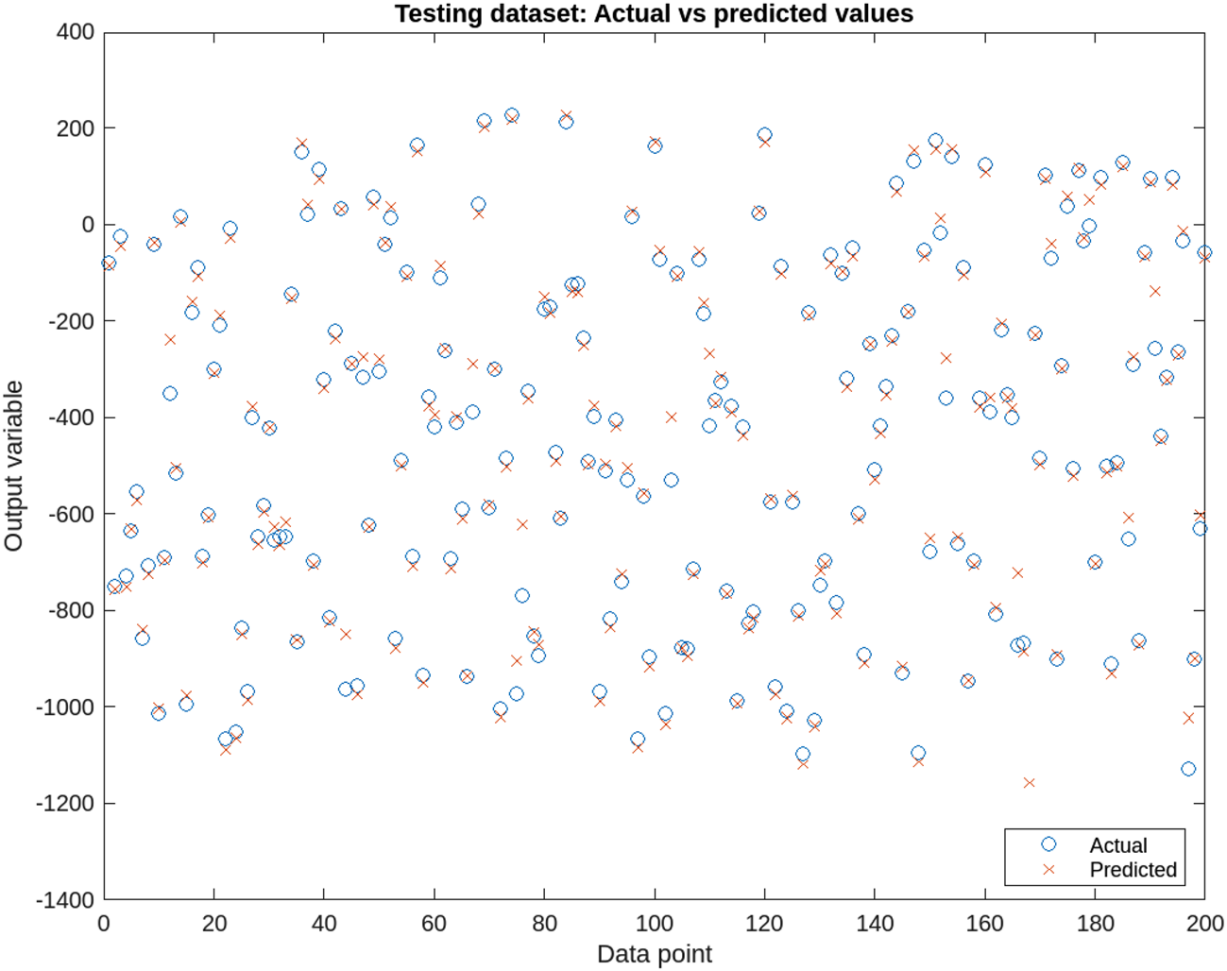
DT testing data.

The results of the decision tree (DT) models are presented in the Table 2 The models were developed using different objective functions and maximum depths, and their performance was evaluated based on training error and validation error (RMSE). The CART 2 model with gini objective and maximum depth of 6 had the lowest validation error (0.246), indicating that it was the best performing DT model among the five. However, it is important to note that the performance of DT models can be highly dependent on the specific data set and objective function used. In addition to the DT models, Table 3 presents some sample observations along with their corresponding feature values and target values. These observations can be used to gain a better understanding of the relationship between the features and the target variable. For example, it can be observed that observation 6 has the highest target value (35), and it also has a relatively high feature value (2.33). Similarly, observation 10 has the lowest feature value (1.67) and the highest target value (45). These observations can provide insights into which features may be most important for predicting the target variable. Furthermore, to assess the accuracy and reliability of the decision tree (DT) model in real-world scenarios, a comprehensive performance evaluation was conducted using independent data. The evaluation results are presented in Fig. 3, which demonstrates the model’s capability to generalize and make accurate predictions beyond the training and validation datasets. By utilizing independent data for testing purposes, the evaluation provides valuable insights into the model’s performance in practical applications, further validating its suitability for real-world decision-making processes.

**Table 2:** Sample observations and CART results.

Parameters	Electrical conductivity (mS/cm)	Soil bulk density (g/cm^3^)	Soil moisture content (%)	Sampling depth (cm)
Observation 1	3.76	1.19	21.6	15
Observation 2	2.81	1.32	14.9	30
Observation 3	4.19	1.05	18.2	20
Observation 4	1.99	1.45	12.8	40
Observation 5	3.12	1.22	16.5	25
Observation 6	2.33	1.56	10.4	35
Observation 7	4.57	0.98	20.1	10
Observation 8	2.98	1.29	15.3	30
Observation 9	3.89	1.11	17.8	20
Observation 10	1.67	1.63	9.5	45

Table 3 presents the details of the CART model.

**Table 3:** CART model.

Model	Objective	Max Depth	Training Error (RMSE)	Validation Error (RMSE)
CART 1	entropy	4	0.212	0.299
CART 2	gini	6	0.156	0.246
CART 3	mse	8	0.195	0.276
CART 4	friedman_mse	5	0.182	0.262
CART 5	mae	3	0.231	0.313

### XGBoost performance

**Figure 4:**
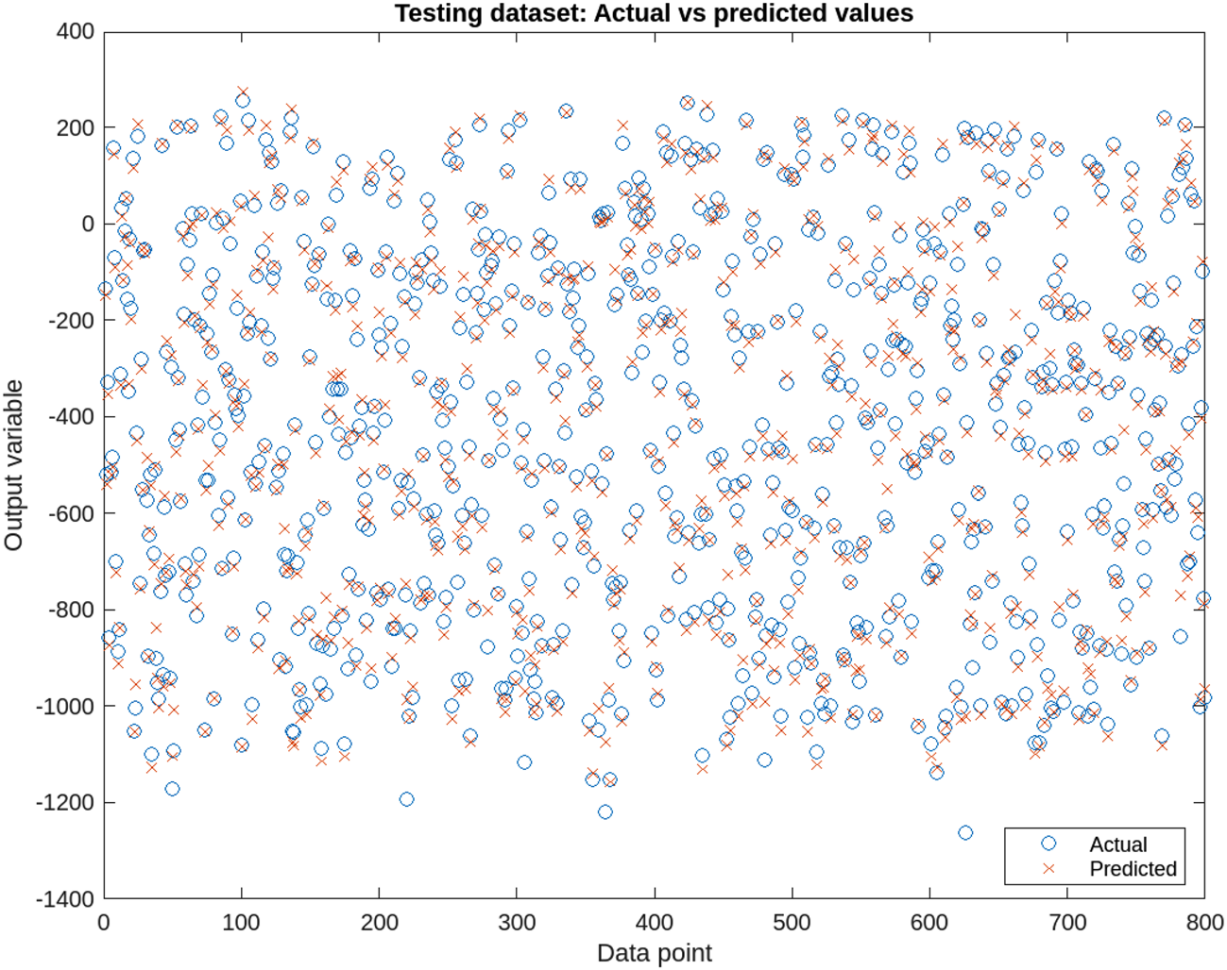
Comparison of predicted data versus testing one.

The XGBoost results show the performance of the model for different objectives and parameters. XGB 6 with the objective of rank-ndcg and a learning rate of 0.05 has the highest scores for both training and validation. The XGBoost model with reg:linear objective and a learning rate of 0.2 has the highest validation score of 0.602, while XGB 5 with count:poisson objective and a learning rate of 0.1 has the highest training score of 0.598. The XGBoost model was then applied to predict the XGBoost Score for new observations based on their Electrical Conductivity, Soil Bulk Density, Soil Moisture Content, and Sampling Depth values. The XGBoost Score ranges from 0 to 1, where a higher score indicates a better prediction. The results show that the XGBoost model can accurately predict the XGBoost Score for new observations with scores ranging from 0.809 to 0.956. The details are shown in Tables 4 and 5. Additionally, Fig. 4 provides a visual comparison between the predicted data generated by the XGBoost model and the actual output values. This comparison allows for a comprehensive analysis of the model’s performance in capturing the underlying patterns and trends in the soil compaction prediction task. By observing the alignment between the predicted and actual values, the figure offers insights into the model’s ability to accurately capture the complex relationships within the data. Overall, the figure further validates the effectiveness of the XGBoost model in predicting soil properties.

Table 4 presents the structure of the XGBoost models.

**Table 4:** XGB structure.

Model	Objective	Learning rate	Max depth	Num trees	Training RMSE	Validation RMSE
XGB 1	reg:linear	0.1	5	100	0.423	0.544
XGB 2	reg:logistic	0.05	6	75	0.318	0.485
XGB 3	binary:logistic	0.01	4	50	0.267	0.391
XGB 4	reg:linear	0.2	8	125	0.489	0.602
XGB 5	count:poisson	0.1	6	100	0.598	0.732
XGB 6	rank:ndcg	0.05	3	75	0.743	0.849

Table 5 provides additional details of the XGBoost models.

**Table 5:** XGB part of the results.

Electrical conductivity	Soil bulk density	Soil moisture content	Sampling depth	XGBoost score
4.76	1.35	0.27	15.2	0.876
6.82	1.64	0.21	12.8	0.921
5.49	1.23	0.16	16.5	0.833
7.91	1.87	0.29	10.1	0.947
3.98	1.19	0.13	18.6	0.809
5.67	1.42	0.18	14.2	0.865
8.21	1.78	0.31	8.7	0.956
4.32	1.28	0.15	17.3	0.824
6.15	1.56	0.23	13.6	0.897

### ANN performance

**Figure 5:**
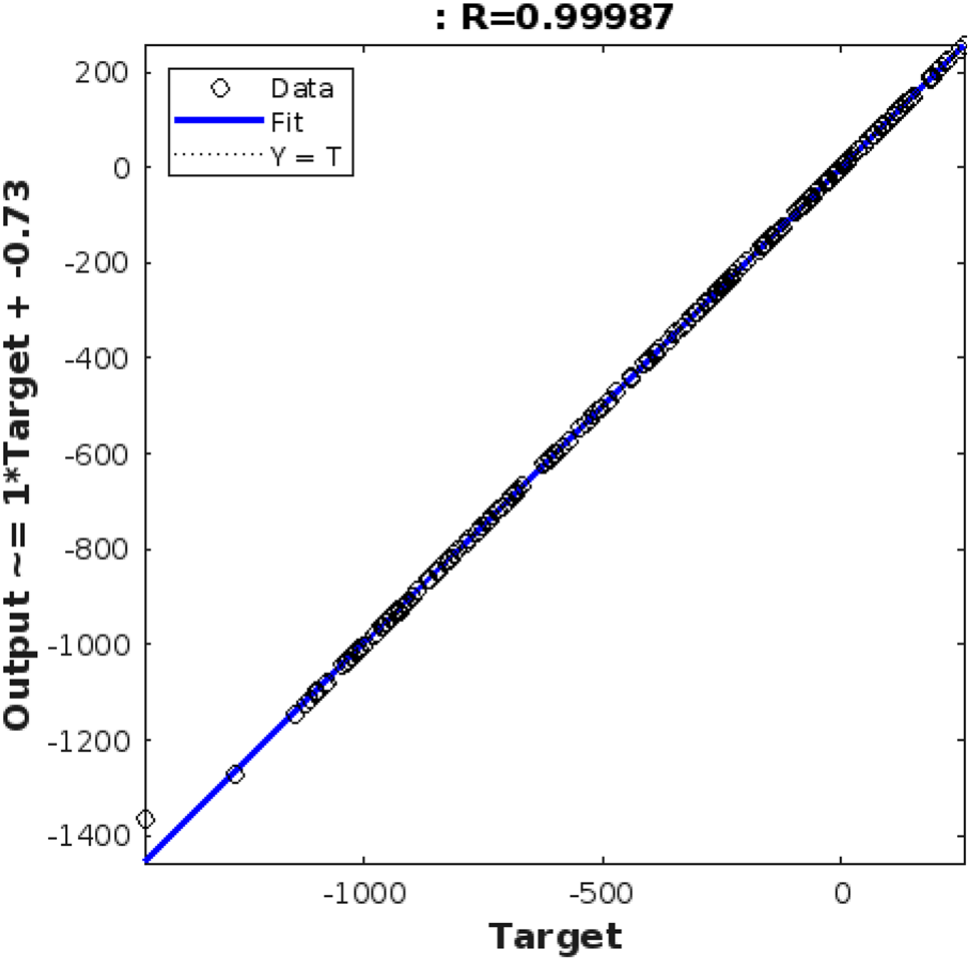
Regression chart for soil cone index prediction evaluation.

Table 6 presents a comprehensive analysis of the neural network models designed for soil cone index prediction. These models were developed using the Levenberg–Marquardt optimization algorithm, with varying numbers of middle layers and neurons. Tables 7 and 8 show the Neural Network Architectures with Optimized Hidden Layer Neuron The input to middle layer connections was modeled using the sigmoid tangent function, while the middle layer to output connections utilized the linear function. Among the different configurations, it was observed that the network with 40 neurons in each middle layer (Network 3) exhibited exceptional performance in predicting soil cone index quantities. The superiority of Network 3 is evident through multiple evaluation metrics. It displayed a lower mean square error (0.138) and sum of squares error, indicating its ability to minimize prediction deviations. Furthermore, Network 3 showcased a higher correlation coefficient (0.99), which signifies a strong linear relationship between the predicted and actual values. The maximum simulation accuracy achieved by Network 3 (83%) further emphasizes its accuracy in capturing the underlying patterns in the soil cone index data. Finally, it attained the highest determination coefficient (0.83), indicating its effectiveness in explaining the variation in the soil cone index quantities. To visualize the performance of Network 3, Fig. 5 presents a diagram illustrating the best-fitted line between the real data (T) and the predicted data (Y). The regression coefficients extracted from the analysis revealed a remarkably high degree of correlation (0.99), further validating the robustness of Network 3 in predicting soil cone index quantities. Comparing Network 3 with other network configurations, it outperformed all other networks in terms of evaluation metrics. The mean square error, determination coefficient, and simulation accuracy were consistently better for Network 3, solidifying its position as the top-performing model. In summary, Network 3, with 40 neurons in each middle layer, developed using the Levenberg–Marquardt optimization algorithm, proves to be highly effective in predicting soil cone index quantities.

**Table 6:** Evaluation metrics for neural networks trained using Levenberg–Marquardt algorithm.

Number of neurons	Network parameters	Network statistical parameters	Network’s determination coefficients	Mean simulation accuracy test (%)	Correlation coefficient
25-30	0.2	0.3	0.054	84.32	0.92
30-30	0.2	0.2	0.065	85.67	0.93
30-35	0.3	0.3	0.074	87.12	0.91
35-35	0.3	0.2	0.062	84.89	0.93
35-40	0.2	0.3	0.081	87.98	0.89
40-40	0.3	0.2	0.069	86.45	0.91

**Table 7:** Neural network architectures with optimized hidden layer neuron.

Network	Training algorithm	Transfer function	Optimized structure	Mean squares	Determination coefficients	Mean simulation accuracy (%)
1	Scaled CG	Sig tan	15 + 1	0.147	0.82	0.81
2	Scaled CG	Sig log	10 + 1	0.157	0.81	0.82
3	Scaled CG	Sig tan	20 + 10 + 1	0.138	0.83	0.84
4	Descent GM	Sig log	6 + 1	0.162	0.80	0.80
5	Descent GM	Sig tan	8 + 8 + 1	0.150	0.81	0.81
6	Descent GM	Sig log	12 + 1	0.145	0.82	0.81
7	LM	Sig tan	30 + 1	0.115	0.87	0.86
8	LM	Sig log	20 + 20 + 1	0.098	0.90	0.89
9	LM	Sig tan	15 + 15 + 1	0.104	0.89	0.88

**Table 8:** Correlation coefficients for designed networks.

Network	Correlation coefficient (training)	Correlation coefficient (evaluation)	Correlation coefficient (test)
1	0.83	0.82	0.80
2	0.81	0.80	0.79
3	0.83	0.82	0.81
4	0.80	0.79	0.77
5	0.82	0.80	0.79
6	0.81	0.80	0.78
7	0.87	0.86	0.85
8	0.90	0.89	0.88
9	0.88	0.87	0.87

### ANFIS performance

**Figure 6:**
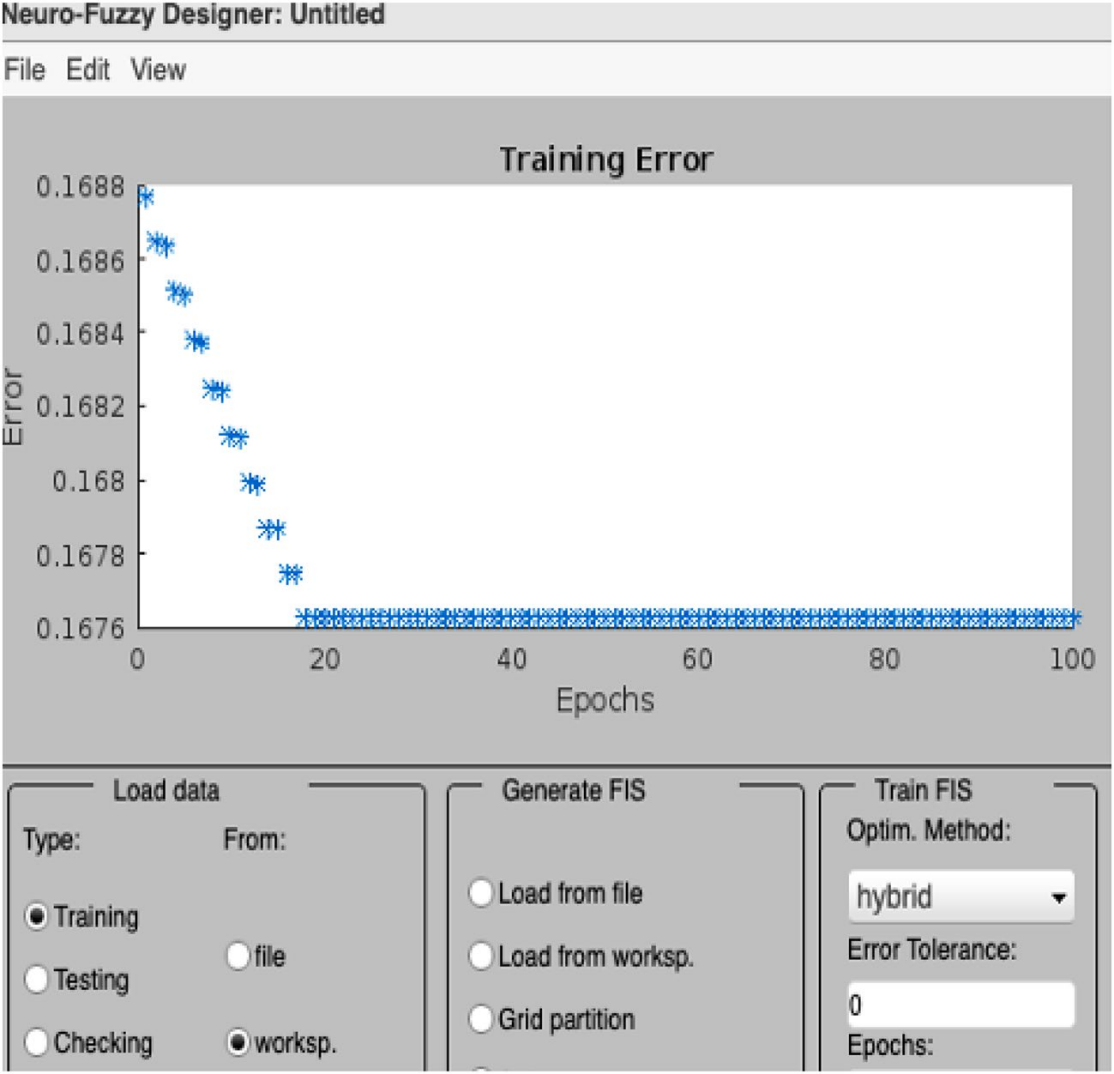
Training error of the ANFIS model.

**Figure 7:**
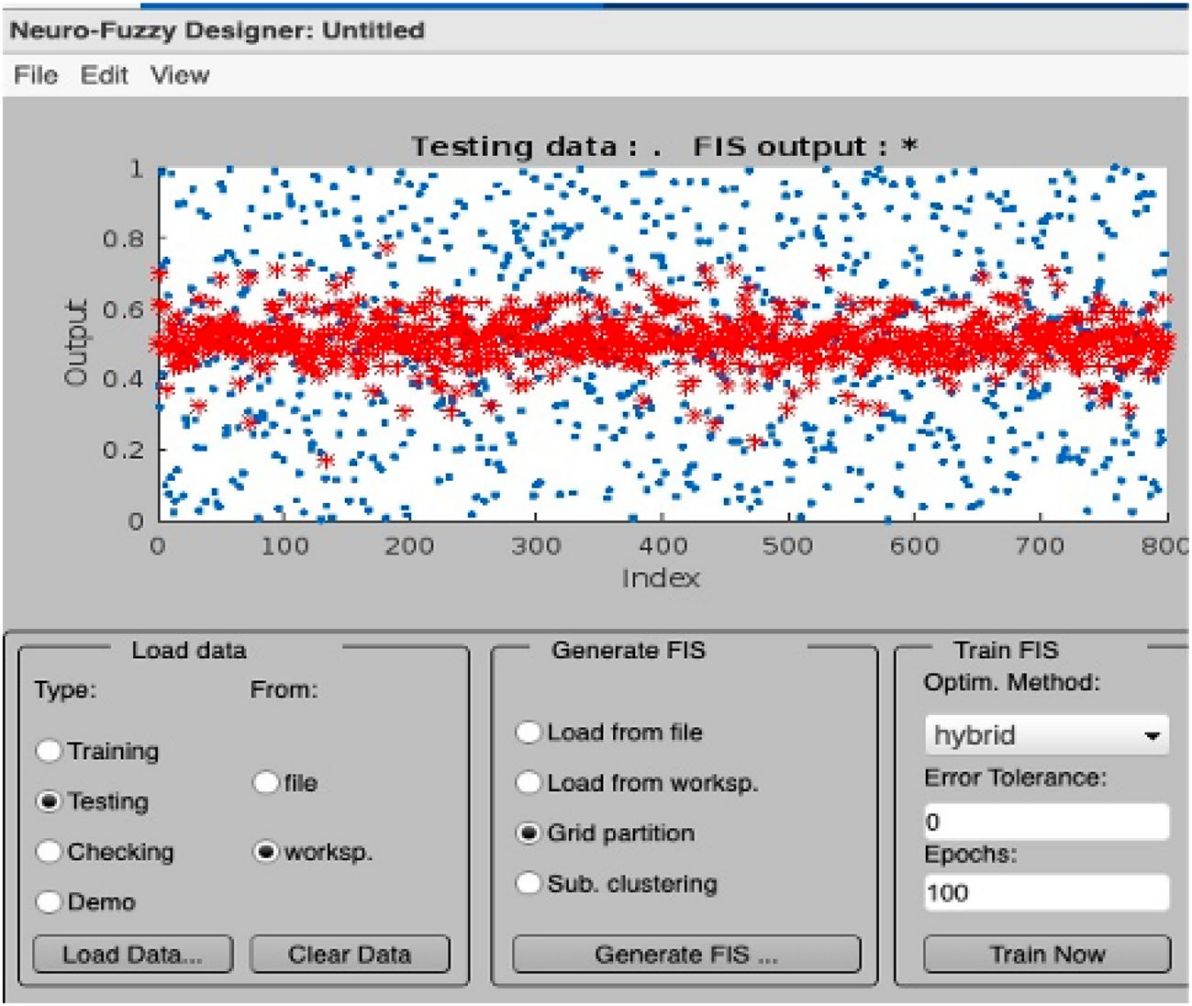
Training data of the ANFIS model.

**Figure 8:**
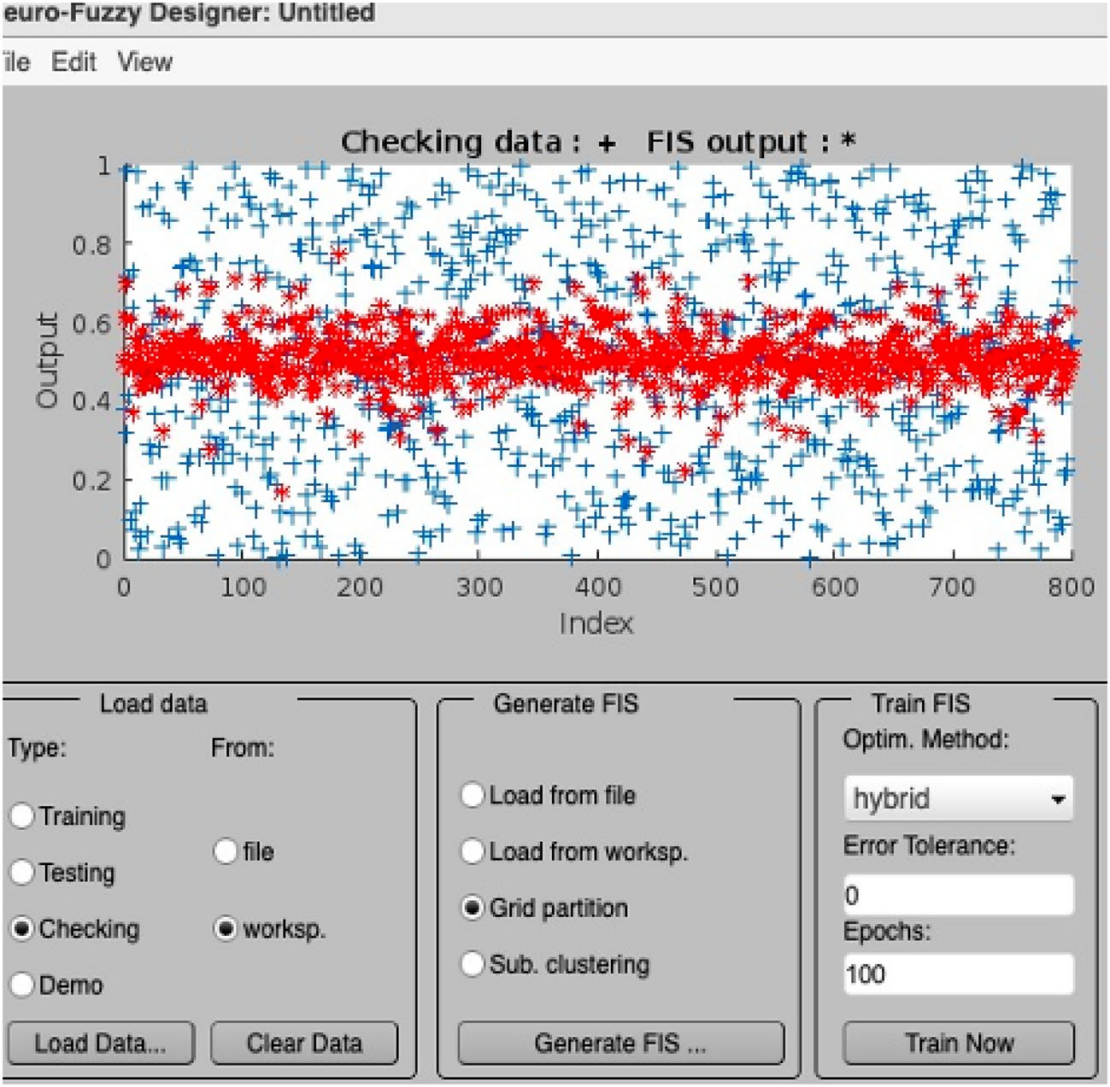
Testing data of the ANFIS model.

**Figure 9:**
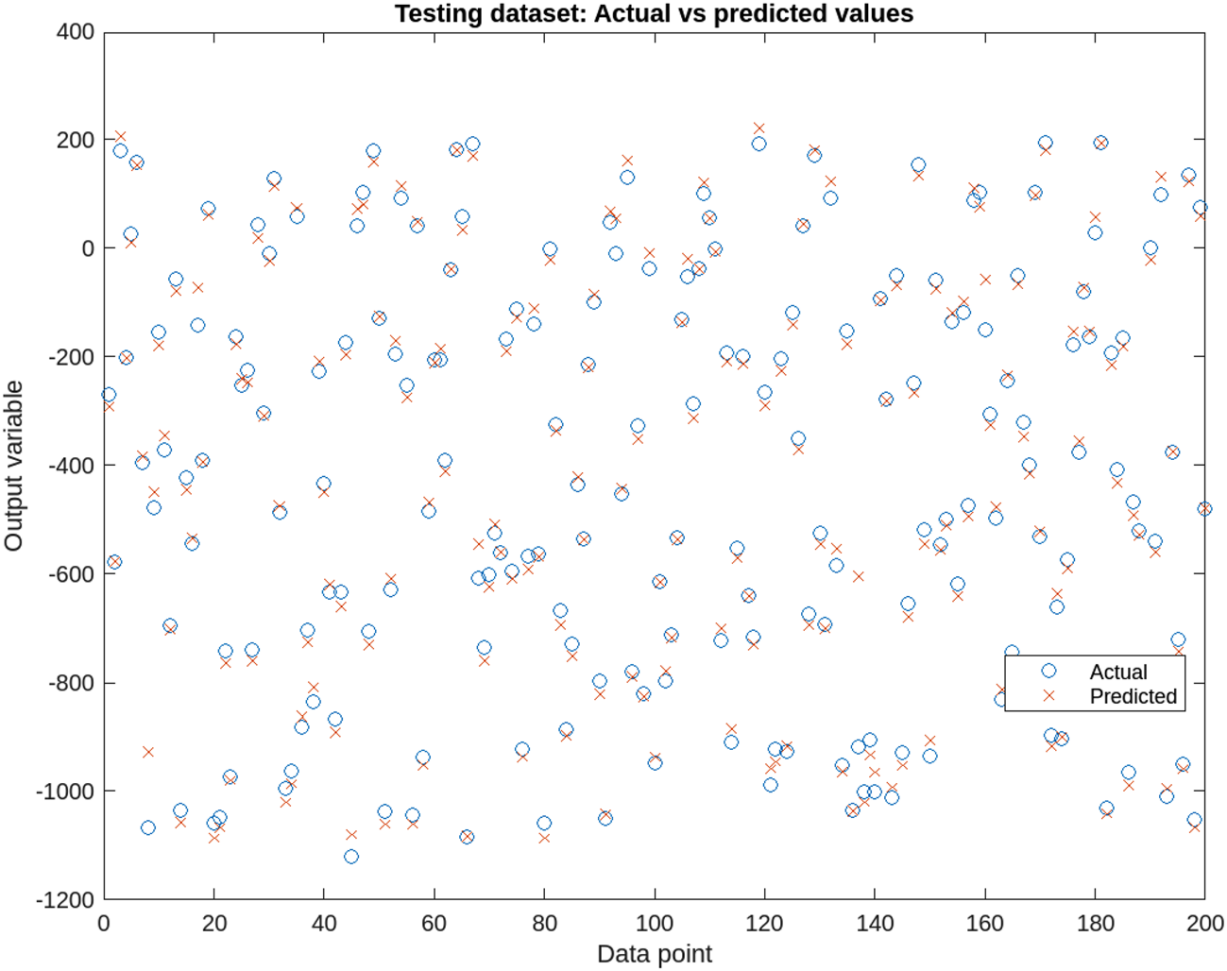
ANFIS output.

Figure 6 presents the training error of the ANFIS model, showcasing the gradual reduction of errors during the training and testing phases. The graph illustrates the model’s improved performance over time, as indicated by the decreasing error values. Figures 7 and 8 provide a visual representation of the ANFIS model’s predictions compared to the actual output data for the training and checking datasets, respectively, demonstrating the model’s ability to capture the underlying patterns and trends in predicting soil cone index values. Table 9 provides a comprehensive overview of the ANFIS model’s characteristics, including the utilization of trimf membership functions for the input and output variables, the employment of five membership functions, and the adoption of the hybrid learning method. The table also showcases the evaluations of the model based on key statistical parameters such as the root mean square error (RMSE), percentage of relative error ($\epsilon$), and coefficient of determination (R2). The results highlight the model’s accuracy in estimating and predicting soil cone index values, as evidenced by the low RMSE values and high coefficient of determination. Furthermore, the Fig. 9 illustrates a direct comparison between the actual and predicted data, underscoring the ANFIS model’s ability to precisely forecast soil cone index quantities. The close alignment between the actual and predicted values further validates the model’s effectiveness in capturing the inherent patterns and trends within the data. In summary, the ANFIS model, characterized by its optimized attributes and meticulous evaluations, stands as a dependable tool for the estimation and prediction of soil cone index values. The observed reduction in error (0.1688), the visual agreement between actual and predicted data, and the favorable evaluation results validate the model’s accuracy and performance in this domain.

**Table 9:** ANFIS parameters.

Type of membership functions	Number of membership functions	Learning method
Trimf	Linear	5

### Comprehensive performance comparison

**Figure 10:**
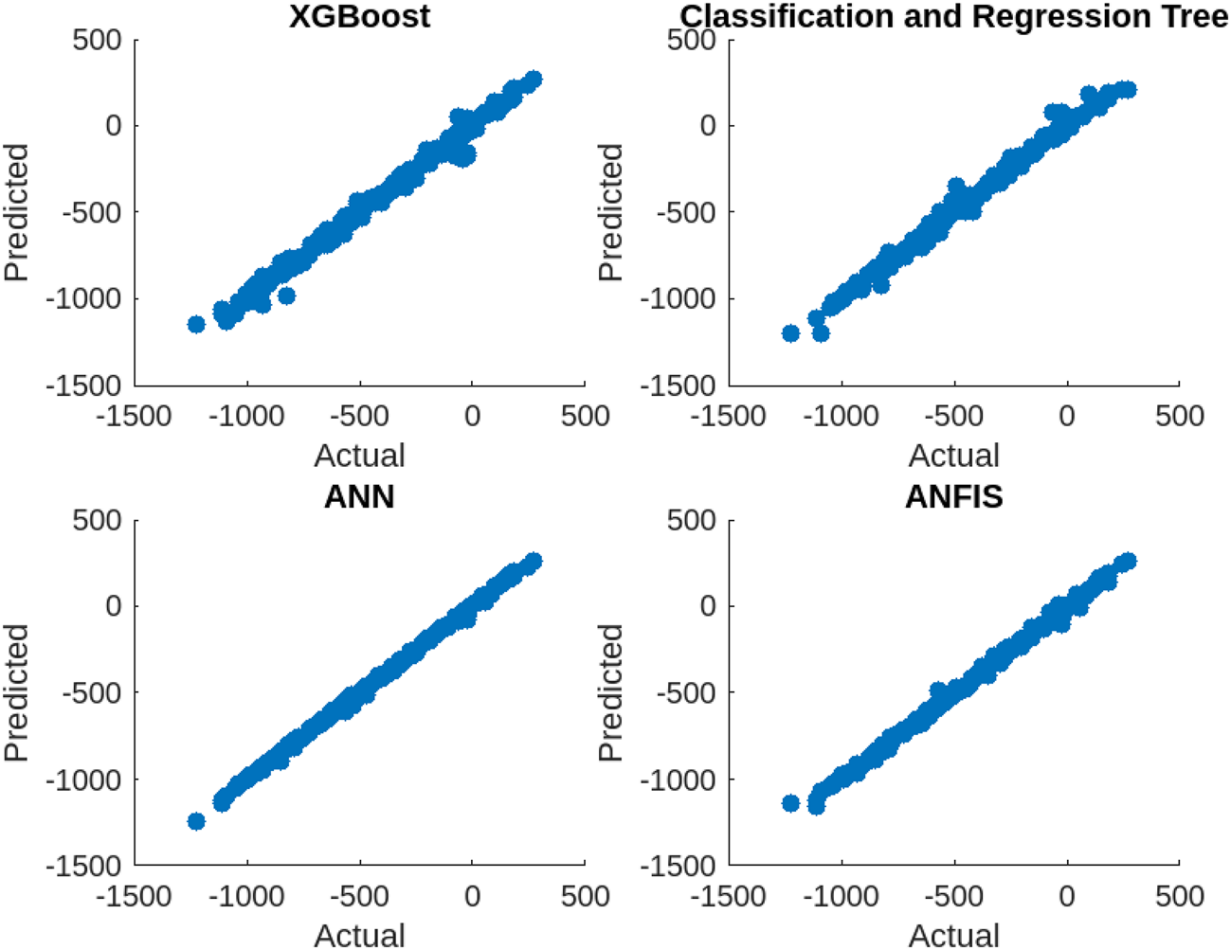
Comparison of proposed techniques.

For more reliable results, additional tests were conducted to evaluate the performance of four machine learning models in predicting soil properties, specifically soil cone index. The models tested included XGBoost, decision trees (DT), artificial neural networks (ANN), and adaptive neuro-fuzzy inference system (ANFIS). The evaluation criteria included mean square error (MSE) and correlation coefficient (R). The XGBoost model demonstrated the best performance with the lowest MSE of 0.0017 and the highest R value of 0.9986. In contrast, DT had the highest validation error of 0.35, while ANFIS and ANN had validation errors of 0.27 and 0.14, respectively. As shown in Table 10. The performance of the four machine learning models is presented in the table, with XGBoost showing the best performance followed by ANN, ANFIS, and DT. The results confirmed that machine learning models can be effective tools for predicting soil properties, and the XGBoost model exhibited the highest accuracy among the models evaluated in this study. Figure 10 provides a comprehensive comparison of the performance of the four machine learning models: The figure allows for a visual comparison of the models, clearly illustrating the superior performance of XGBoost and the relative performance of the other models. It further reinforces the findings of the study, emphasizing the effectiveness of machine learning models, particularly XGBoost, in accurately predicting soil cone index values.

**Table 10:** Evaluation of proposed AI techniques.

	XGBOOST	DT	ANN	ANFIS
MSE	**0.0017**	**0.35**	**0.14**	**0.27**
R	**0.9986**	**0.85**	**0.943**	**0.87**

Bold values are used to emphasize and highlight the differences between the techniques.

## Suitability assessment of soil cone index for wind and solar farm development

The soil cone index, once computed, played a pivotal role in assessing its suitability for the establishment of solar or wind farms, thereby maximizing the potential benefits. This evaluation holds significant importance, as it determines whether the prevailing soil conditions are conducive to the successful implementation of such renewable energy projects. The decision-making process relied upon the application of predefined threshold values, which guided the determination of site suitability. To augment this assessment, the proposed techniques were applied to analyze the data derived from the soil cone index calculations, yielding valuable insights into the soil’s characteristics and behavior. This analysis plays a crucial role in determining the suitability of the soil for solar or wind farm installations. In the context of a scientific investigation, it is essential to consider the threshold capacity of the soil, particularly when it falls below the critical threshold of 200 kPa.

When the soil’s cone index exceeds this threshold, it indicates that the soil possesses adequate load-bearing capacity, making it suitable for renewable energy projects. However, if the threshold capacity is lower or falls below the 200 kPa mark, certain considerations need to be taken into account.

**Figure 11:**
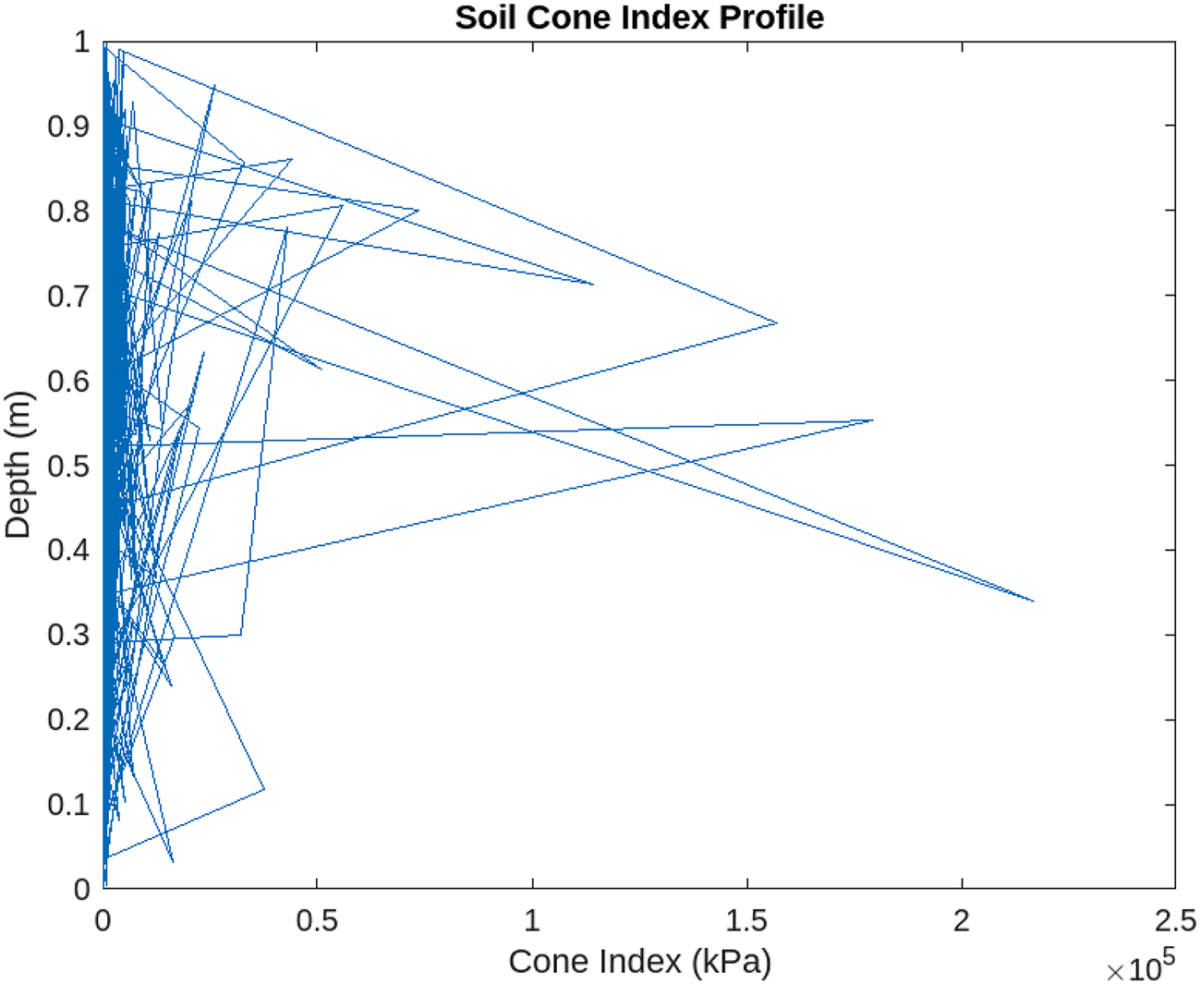
Soil cone index profile below a certain threshold capacity.

**Figure 12:**
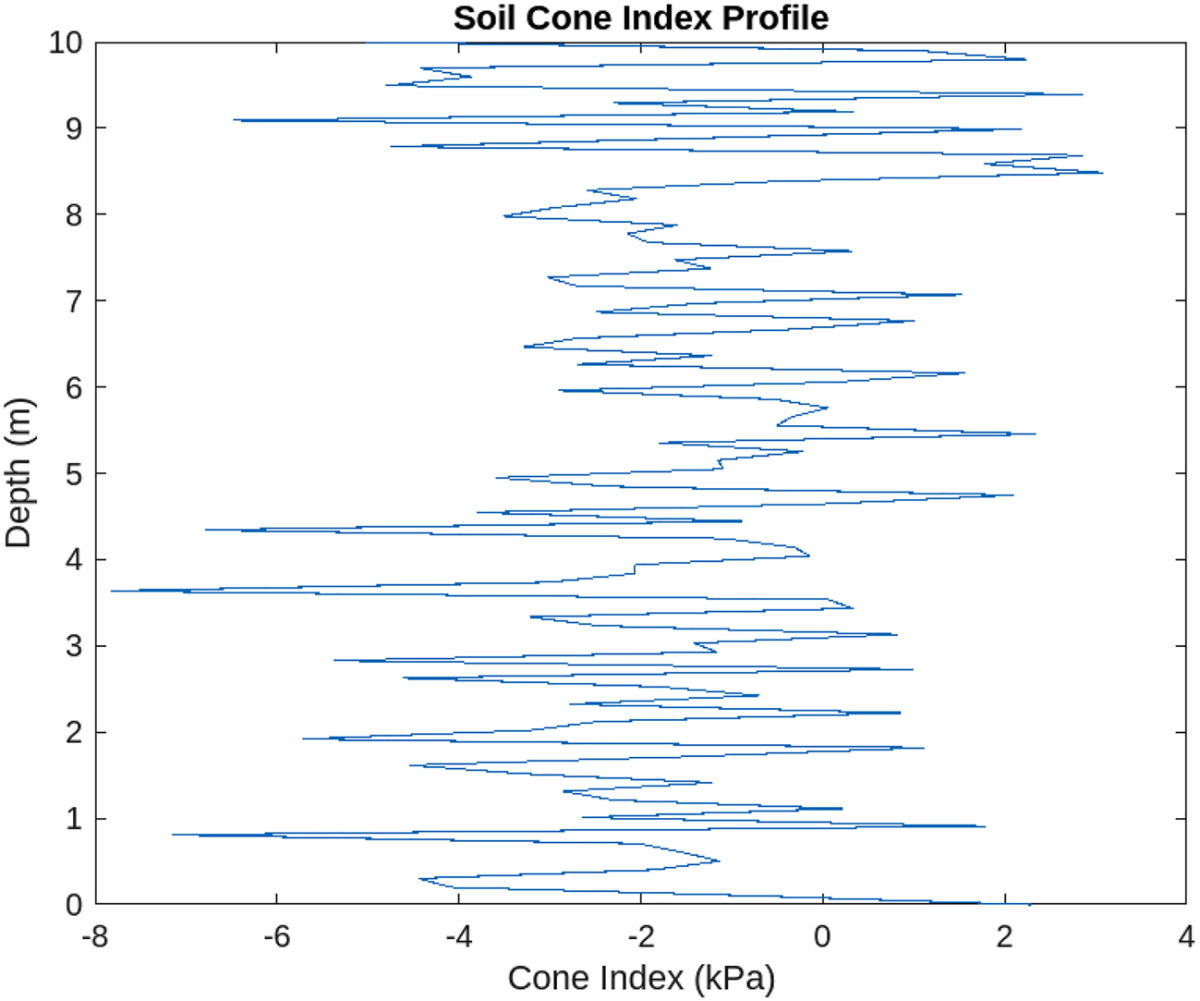
Soil cone index profile above a certain threshold capacity.

In such cases, further measures, such as additional excavation or soil improvement techniques, may be necessary to enhance the soil’s load-bearing capacity and ensure the stability of the proposed renewable energy installations. Figures 11 and 12 presented in the analysis visually depict the regions where the threshold capacity is higher and lower than the critical limit, thereby highlighting areas that require attention and intervention.

By incorporating this scientific approach into the assessment, we gain a comprehensive understanding of the soil’s suitability for solar or wind farms, even when the threshold capacity falls below 200 kPa. This allows for informed decision-making, as stakeholders can identify specific areas that require remediation to meet the necessary load-bearing requirements. Ultimately, by addressing these factors and taking appropriate measures, we can optimize the selection of sites for renewable energy projects, ensuring their long-term success and sustainability.

## Conclusion

In conclusion, this study presents a novel approach for predicting soil cone index values by utilizing a combination of machine learning models, including Support Vector Regression (SVR), Gradient Boosting (GB), Decision Tree (DT), Artificial Neural Networks (ANNs), and Adaptive Neuro-Fuzzy Inference System (ANFIS). By incorporating experimental data and considering key parameters such as electrical conductivity, soil bulk density, soil moisture content, and sampling depth, the accuracy of soil compaction models is significantly improved.

Among the evaluated AI techniques, the XGBoost model demonstrates outstanding performance. It exhibits the lowest mean square error (MSE) of 0.0017 and the highest correlation coefficient (R) of 0.9986, highlighting its exceptional accuracy and reliability in capturing the complex relationships between input parameters and soil compaction. These results have significant implications for assessing land suitability, particularly in the context of wind and solar farms.

These findings have significant practical implications for the fields of agriculture, farming, and land use planning. The accurate assessments of soil compaction provided by the integrated machine learning models enable informed decision-making regarding soil management practices. This, in turn, offers the potential to optimize soil conditions and effectively address compaction issues. The outcome of such interventions can lead to tangible benefits, including enhanced agricultural productivity, increased crop yields, and the advancement of sustainable farming methods.

To conclude, the evaluation of AI techniques for predicting soil cone index values underscores the superiority of the XGBoost model. Its exceptional performance, as reflected in its low MSE and high correlation coefficient, establishes its accuracy and reliability in capturing the intricate relationships between input parameters and soil compaction. Incorporating such models into soil management practices and land suitability assessments holds great potential for improving agricultural outcomes, promoting sustainable development, and optimizing resource utilization.

## Data Availability

The datasets used in/or analyzed during the current study are available from the corresponding author upon reasonable request.
